# Are the Public Competent to Judge What System of Medicine Is Most Desirable, or What Practice They Should Adopt?

**Published:** 1873-02

**Authors:** T. D. Washburn

**Affiliations:** Hillsboro, Ill.


BUFFALO

### Medical and Surgical Journal.

VOL. XII. FEBRUARY, 1873. No. 7

### Original Communications.

:o:

ART. I.—*Are the Public competent to judge what system of medicine is most desirable, or what practice they should adopt?* By T. D. WASHBURN, M. D., Hillsboro, Ill.

These are questions urged home upon every adult, single or married, as the necessity for a choice arises. Most persons reaching the age of maturity have their minds pretty well made up before the emergency of sickness or accident arrives.

How is this judgment formed? What element or force decides? Is it the *ability* of those representing the system? Is it fashion? Or is it a conclusion only reached after weighing well the *merits* of the system? All these occasionally and more than these. The same considerations rule in this matter as in selecting a place of worship, or a lawyer; we are governed by our friends, our *social* surroundings, our parents, or early associations; we make the selection more frequently without weighing any evidence, without reflecting on the result of a false step or a wrong selection.

We are influenced by the *man*, his personal presence, his social qualities, his culture, his politics, his religion, even, or *want* of it; and once chosen, or accustomed to a certain "ism" or "pathy," we are likely to remain. In the popular language of the day, "we go it blind," and though we fall into the ditch, we still cling to the same leader. It's astonishing how some worship age. They have a perfect abhorence of anything *new*. They believe in their greatgrand-father's politics, his knee-buckles, his wooden mould-board plow, and his old-fashioned doctor, with his roots, herbs, lancet and calomel. They are like old national patriots, "our country, right or wrong," our father's doctor, "right or wrong." They belong to that class known mostly in the rural districts of Tennessee that are still voting for Jackson, and havn't made up their minds that the negro is *free*. They reject facts that do not have the smell of age on them. Their meat must be *salt* or *tainted*.

But it is time for us to inquire what influences are at work to lead the masses away from regular medicine? Chiefly *two*. The zealous advocates of *false* systems, or dogmas, stationary and itinerant, and the nostrum or patent medicine system. The former exerts a *personal* influence and pushes itself into prominence and notoriety by cheek, pluck and assumption, and various means, honest and dishonest; the other through the press, poster, circular, almanac, deceives most basely by imposing testimonials, *made up affidavits* and most willful lying. The innocent are deceived, the public gulled and swindled, and the most absurd notions and views made current. Look at Prof. Leonidas Hamilton using a *whole* page of the largest and most popular religious weekly to advertise his wonderful self and wonderful wares, mixing his hypocritical piety with his medical shams and delusions. Then look at the paper, which has more readers than any two, and shapes and generates the opinions of half the thinking men in the United States, the New York *Tribune*, and behold the *same* thing, a whole page devoted to Leonidas Hamilton, with paid puffs, and paid testimonials, and we need not be surprised that a goodly number are led astray.

True, the innate superstition of mankind, the love of the marvelous, and the willingness to be deceived, manifest in all generations since the first quack doctor, the Devil, made his appearance, undoubtedly makes it easier for many to accept *error* as truth, the false for the real, and confuses and bewilders the honest mind. Then, the multiplied forms, the universality of nostrums, the very air we breathe seems pregnant with them. No newspaper, periodical or journal is exempt. They come as wrappers; they are left to try, to sell; they walk, they ride, they almost fly; they are dropped in the wagon and buggy when you come to town. They are painted on fences, thrown from balloons, travel on steamboats and cars. Strange if all these should not exert some influence.

But, gentlemen, those that are *for* us are more than those against us. I speak of our own country. Ignorance is beginning to yield to knowledge, even in medicine. Truth, with a *fair* contest with error, will always be successful. The false must succumb. The common school is a foe to quackery, and though the press may give currency and advertise their nostrums and dirty items, with the tinsel of false medical doctrine (for which they are well paid), still it carries a partial antidote. Its columns are open to discussion, and its very freedom and variety of topics stimulate thought, and this is an enemy to shams.

Science, in all aspects, is arousing from its slumbers, and vigorously and loudly asking the reason *why*, and quackery and dogmas can't answer.

Our own journals are alive and awake to this matter, and an experience of near half a century has not been without its results. The animus, habits and status of irregular medicine has become pretty well known. Its base assumptions, unscrupulous means, false position and transparent weakness are every day more apparent. All candid issues have been fairly met. The good they have accomplished has been placed to their credit.

We have frankly canvassed the abuse of calomel and the lancet, and their use has been modified. We have been as diligent as they in exploring the botanic world, and analyzing and detecting its various virtues, and brought far more science and clinical experience to demonstrate its worth or worthlessness.

I challenge them to show a single scientific fact, which they claim, that we do not accept; a single remedy of any virtue we do not acknowledge, or any other practical good known to the medical world, which we do not endorse, appreciate and use.

Likely they will assert that the eclecticism of the present is not the Thompsonianism of yesterday; yes, but it is the *boy* grown to *manhood.* The acorn to an oak. It is a *false* system, and mainly *inimical* to *regular* practice, as it was in its childhood. It has no *creed* of its own, no *principles,* or base upon which it stands. It is

Thompsonian, physio-pathy, eclectic, chemico-rootico, botanico, homo-destructo, or otherwise, to suit the latitude and bias of its friends. Why! in our own county you can see one of its most distinguished lights, with the crude root in one hand and a vial of sugar pellets in the other, ready to serve his patrons with *either* a bountiful, nauseous dose of mandrake and pocoon, of the knock-down and drag-out genus, or some inert, tasteless, worthless stuff. *Two* pellets in a tumbler of water—take seven drops every twelve hours. Ask one of them wherein they differ from the regular practice, and the sum total of their reply is: "We don't give calomel." Even the *class-books* recommended by these schools are the identical ones we use, mostly. They have *nothing* but what they have taken from us. No sect ever gave such *insuperable* evidence of *paucity* of ideas and general stealing as the Eclectics; yet there is no end to their lofty vaunting and bold assumptions.

So too Homœpathy, another irregular gentleman, deserves a passing notice. Its *trade-mark*, "infinitisimals," is lost. Some are manly enough to admit they rarely use the *higher* dilutions, and now and then give allopathic doses. And, while we must concede, they are more learned than other *irregulars*, and they assume a kind of platform or declaration of principles, still it presents no candid claim to science, and they adhere no more closely to its founder, Hahnemann, than the Eclectics do to Thompson. It is a system of "nihilism,"—*do nothing—nature, hygiene* and *diet.* But grant their remedies have some virtue, the simplest tyro knows that prescribing for *symptoms* is not necessarily prescribing for disease, but this is one of the *principal* factors with them. Its history for a quarter of a century has been a curious one, changing like a kaleidoscope both its doctrine and its practice, driven from one position to another, and ready to die in the last ditch; tested by the most eminent physicians in hospital and private practice, at home and abroad, but invariably wanting and unable to establish its claims. Not a school, college or university, and but few practitioners in the home of its nativity; it has come over to the universal Yankee nation to make its last stand, play its pranks, with other mountebanks and charlatans, and meet eventually its final overthrow.

I should deem myself culpable to speak disrespectfully of Homœpathy, did they adhere strictly to their principles. But when professing to know a *better* way, they boldly give regular medicine in regular doses, to keep the patient from slipping out of their hands, or to relieve his pains, they are more blameworthy than their less enlightened neighbors, the Eclectics, who hold to their patient and their practice to the bitter end. To pretend to cure with "little pills" when resorting to two-grain quinine pills and plenty of them, looks very much like a *swindle*, to use no harsher epithet.

But, with all our censure for pretense and false systems, medical dogmas and quackery in many of its popular forms, we admit that it has had a good effect on regular medicine. Thompson, by his antagonism, gave it a healthy stimulus. He drew attention to some of its abuses, the effect of which was to remedy the same. But in doing this, he inflicted a far greater evil on society. An ignoramus himself, he was poorly qualified to give law and being to a new system. But the *hotch-potch* came, and the lazy Yankee took to it, as a duck takes to water. A man who had mastered the spelling book was ready to acquire and practice the system. Men rushed from the workshop and the farm to rise suddenly to eminence as a doctor. The sewing machine and insurance business had not yet put in an appearance.

His system of cheap medicine, false in theory and in practice, only required six weeks to master in all its details, while anatomy alone cannot be learned in twice that time. But my Eclectic friend replies : "We have outgrown Thompsonianism. We do not confine our practice to the use of red pepper, lobelia, and steaming." I admit they have done a good deal with indigenous botany, and added several items to the materia medica, and aroused an interest among ourselves which might have lain dormant some time. But they have been and still are the hot-bed of charlatanism and quackery in its worst forms.

They have placed a premium on ignorance that years cannot eradicate. They foster superstitution, and thus put up barriers to improvement. Their low standard of requirement invites every dunce in the country to come into fellowship, and thus the masses lose confidence in all systems. Of late years, an M. D. from one of their institutions has seemed to possess considerable attraction to them. It has only proven the extreme rottenness of the whole concern. Philadelphia, anxious to monopolize this business, for a few dollars granted these diplomas by letter; one did not even have to put in an appearance. The State saw its mistake and annulled their charter. But Ohio has a factory not much behind: at present a few weeks residence are necessary to secure the coveted prize; but no one can tell how soon their degrees will be a drug in the market and sell for less than a bankrupt stock. Yet these same doctors, eclectics of the nineteenth century, arrogate to themselves the proud position of *leaders*, progressive men, in advance of all others, genuine, pure and unadulterated, while the regulars are "old fogies," behind the age,—so prejudiced they will not receive *eclectic* light; "calumy doctors," *forty* years behind the times. Such stuff they daily thrust on the people, and many are their dupes. But what are the *facts?* In what respect are they first, or in *advance*, or pure, or genuine and progressive? After fifty years of noisy declamation among a *gullible* people, how is it? What do statistics say?—*four* eclectic medical schools in the United States against *sixty-two* regular, and *six* homœopathic, with a still greater disparity of journals.

Does this look like outstripping the "old school?" Are these symptoms of progress and radical improvement? No, gentlemen, the assertion is not true. They are fully conscious they are far enough in the rear. But they think by keeping up a din of noise and loud hurrahs, they will prevent their patrons from discovering their weakness. Let them howl; we need not be alarmed. Let them keep up appearances; have a few colleges, a few journals, and half a dozen fairly qualified professors, a few sore heads that have left the regular profession, yet they know that no system but the *regular* is worth a straw, or has any chance of success. The whole history of medicine proves this, all statistics corroborate it. The rapid growth of knowledge, the new fields of science. Free and fair discussion on the rostrum and through the press forbid any other opinion. The *broad* platform on which regular medicine is built, its catholic spirit and toleration of wide views and practice give no occasion for any other system. The very fact that all others are not what their friends represent them, but false in theory, unsound and absurd in practice, and uncalled for by the public, give us the strongest assurance they will go to the wall. The elements of dissolution are in them. All genuine reforms must be founded on some grand *principle* as a rallying point, some genuine *good* to be obtained, or some well defined *evil* to be removed; but this has neither. Its extinction is only a matter of time.

To return then to our starting point. Are the public competent to judge in regard to the system and practice they need? or as likely to judge correctly as in most other matters? I am inclined to concede they are. When we see how strangely they err in other matters, how often they are duped, how the most wary are caught, and how few are able to divest themselves of cant, bigotry and superstition in some form, it is not a matter of surprise that some who make large pretensions to culture and refinement are found blindly worshipping the most ridiculous and absurd *theories* of medicine, as well as patronizing the most consummate quacks.

They have *one* excuse. The public and the general culture of the world are taught *less* of medicine and disease than any other one thing in the vast range of knowledge which concerns them so directly. They know less of the laws of their own mental and physical constitution than they do of their neighbor's domestic relations or the last problem in social science. They know better how to raise, care for, and preserve the brute creation than their own kind; but these days are fast passing away; a better era approaches. The State (at least Illinois) has discovered the mistake and is attempting to repair it as wisely as possible by requiring Physiology to be placed among the elementary branches of the *free* school, and ere long every child will know something of the laws of *life*. So much for healthy legislation. The next step will be to *label* patent medicines with their real ingredients, and thus *rob* them of their charm; the next, to require every practitioner to give some evidence of his acquaintance with the human system and his qualifications to practice medicine. Gentlemen, in *some* form these things will come, and at no distant day. They will help to eradicate error; they will encourage truth, lessen humbugs, and prepare the masses to find the right way more easily.

We may blame ourselves for a large share of our own misfortunes; causes exist which make our enemies prosper; our "*esprit de corps*" is not what it should be; we have neglected to listen to just criticism; our ethics have been misunderstood; we have been silent without cause when our *good* was evil spoken of; our competitors have taken advantage of every mistake; they study individuals more, books less. In their general practice they rely more on protracted calls, genial stories, and ostentatious nursing; but above all, they talk loud and learned; they always have a *name* for every indisposition; they make frequent allusions to themselves and their wonderful cures. Their cases are usually very *severe,* unless another school is called in, then they are *mild.* They make a conspicuous *show* of business; they are partial to editors, and either furnish locals or have their friends to do it often. They *know* everybody; do business cheaply are not inclined to adopt fee-bills; are all things to all men; rather in advance of the apostle.

Many good qualities they have which often compensate in the public eye for great deficiencies. Here and there gross ignorance crops out, and the greatest blunders are made, but the regular has learned to keep his mouth closed, or the cry of persecution will be raised. But I am entering too much into detail; I only designed to draw the lights and shadows as they are often presented in *living* colors. Some things may be worthy of adoption. Many of this class of practitioners are as honest in their views, as conscientious in their practice, as any *regular.* They are the creatures of circumstances; they were born with such surroundings, and are nowise blameworthy. But that a vast difference exists between the two systems and classes of practitioners, both theoretical and practical, no one will deny.

Perhaps some one may ask, how shall we separate, judge and distinguish the chaff from the wheat, the true from the false, the worthy from the unworthy? It is a legitimate question, and I will briefly attempt to reply and bring my incoherence to a close. A few words first in regard to the "*regular.*" Possibly I may have left the impression that if one selects a "regular" to attend, he professionally, is safe, and may dismiss all anxiety about the result. No! I do not say that; so far as the *system* of medicine is concerned, the regular is the only *reliable* and *safe* one ; but unfortunately in *practice* there is no monopoly of brains in any profession, sect or people. The *system* is founded on the most advanced science in all of its departments, and the best authenticated facts demonstrated by the largest experience ; but the system does not furnish those who embrace it with brains to comprehend it, and like *other* systems is often grossly misrepresented by its ignorant or weak disciples.

True, its standard of qualification is far higher than the others ; but the lame and weak are found in every fold, and with all the safe-guards, and barriers to imposition, more or less enter entirely unqualified, and ere long disgrace themselves and their profession.

The good Book lays down the *true* standard : "By their fruits ye shall know them." "Do men gather grapes of thorns, or figs of thistles ?" Yes, gentlemen, the public will judge us and them *mostly* as they judge other men. They may be deceived for a time by noise, bluster and pretence, but the time is close at hand when the mask of hypocrisy shall be torn off. Cliques, sects and leagues though leagued with evil shall not triumph much longer. The American people and the nations of the earth begin to realize that what "has been *covered* shall be revealed," what "has been hid shall be make known." In government, religion, and medicine, the crooked shall be made straight, and the rough ways smooth. The spirit of enquirv and investigaton is abroad in the land. The time is not far off when the citizen will exercise as much judgment in choosing his doctor as he does his shoemaker, tailor or blacksmith ; we ask no more. They would do it now, but they have not the information in regard to the system and the nature of disease. besides it is only at unequal intervals they are called upon to make choice ; whereas these other things pertaining to our comfort and happiness are of *daily* occurrence. But let *us* see to it that we are worthy and well qualified; let not the greed of *avarice* be the ruling passion. As business accumulates let us not neglect authorities, the council of the master-minds and great workers of the profession. We must bring the most untiring devotion to the task. To succced, we must *love* our profession ; we must study disease at the bedside, on horseback, and from the books. If one course of treatment fails, change the programme; avoid hobbies and pets. Let us investigate the local endemic and individual causes operating to generate diseased conditions; the laws of hygiene, diet, temperature and septic influences should not be ignored and overlooked; we should point out the antidote as well as the cure of disease; we must not enter every professional opinion on our day-book as a prescription, nor devote more thought and anxiety to the *fee-bill* than to the *care* of our patient. In a word, we must aim to keep ourselves abreast of the best teachings and practice of our art; liberal in our views, catholic in spirit, sympathetic, pure, generous, and humane; let us bless the world by our devotion to our profession, and receive that compensation above the paltry sum of dollars and cents, an approving conscience, without which the world, position, influence and affluence are the merest chaff.

